# Assessment of country implementation of the WHO global health sector strategy on sexually transmitted infections (2016-2021)

**DOI:** 10.1371/journal.pone.0263550

**Published:** 2022-05-04

**Authors:** Melanie M. Taylor, Teodora Wi, Antonio Gerbase, Soe Soe Thwin, Sami Gottlieb, Maria Theresa Babovic, Daniel Low-Beer, Monica Alonso, Maeve B. Mello, Naoko Ishikawa, Anne Brink, Joumana Hermez, Ahmed Sabry, Saliyou Sanni, Leopold Ouedraogo, Bharat Rewari, Mukta Sharma, Nicole Seguy, Elena Vovc, Ian Askew, Meg Doherty, Nathalie Broutet

**Affiliations:** 1 Department of Sexual and Reproductive Health and Research, World Health Organization, Geneva, Switzerland; 2 Department of HIV, Hepatitis and STI, World Health Organization (WHO), Geneva, Switzerland; 3 Division of STD Prevention, U.S. Centers for Disease Control and Prevention, Atlanta, Georgia, United States of America; 4 Geneva Foundation for Medical Education and Research, Geneva, Switzerland; 5 WHO Regional Office for the Americas, Geneva, Switzerland; 6 WHO Regional Office for the Western Pacific, Manila, Philippines; 7 WHO Regional Office for the Eastern Mediterranean, Cairo, Egypt; 8 WHO Regional Office for Africa, Brazzaville, Republic of Congo; 9 WHO Regional Office for South-East Asia, New Delhi, India; 10 WHO Regional Office for Europe, Copenhagen, Denmark; University of Alabama at Birmingham, UNITED STATES

## Abstract

**Background:**

In 2016, WHO launched the Global Health Sector Strategy on STIs, 2016–2021 (GHSS) to provide guidance and benchmarks for country achievement by 2020 and four global targets for achievement by 2030.

**Methods:**

A country survey jointly developed by experienced technical personnel at WHO Headquarters (HQ) and WHO regional offices was reviewed and distributed by WHO regional advisors to 194 WHO Member States in September-March 2020. The survey sought to assess implementation and prioritization of STI policy, surveillance, service delivery, commodity availability, and surveillance based on targets of the GHSS.

**Results:**

A majority (58%, 112/194) of countries returned a completed survey reflecting current (2019) STI activities. The regions with the highest survey completion rates were South-East Asia Region (91%, 10/11), Region of the Americas (71%, 25/35) and Western Pacific Region (67%, 18/27). Having a national STI strategy was reported by 64% (72/112) and performing STI surveillance activities by 88% (97/110) of reporting countries. Availability of STI services within primary health clinics was reported by 88% of countries (99/112); within HIV clinics by 92% (103/112), and within reproductive health services by 85% (95/112). Existence of a national strategy to eliminate mother-to-child transmission of HIV and syphilis (EMTCT) was reported by 70% of countries (78/112). Antimicrobial resistance (AMR) monitoring for gonococcal infection (gonorrhoea) was reported by 64% (57/89) of reporting countries with this laboratory capacity. Inclusion of HPV vaccine for young women in the national immunization schedule was reported by 59% (65/110) and availability of cervical cancer screening was reported by 91% (95/104). Stockouts of STI medicines, primarily benzathine penicillin, within the prior four years were reported by 34% (37/110) of countries.

**Conclusions:**

Mechanisms to support improvements to STI service delivery through national-level policy, commitment, programming and surveillance are needed to operationalize, accelerate and monitor progress towards achievement of the 2030 global STI strategy targets.

## Introduction

In 2019, the World Health Organization (WHO) estimated that 376 million cases of four curable sexually transmitted infections (STIs) (chlamydia, gonorrhoea, trichomoniasis, and syphilis) occurred annually [1]. Undiagnosed and untreated STIs can result in adverse reproductive health outcomes including infertility and ectopic pregnancy and adverse birth outcomes such as stillbirth (syphilis) and prematurity/low birth weight [2]. WHO’s Global Health Sector Strategy on Sexually Transmitted Infections for 2016–2021 (STI Strategy) was launched alongside global strategies for HIV and viral hepatitis as priority guidance for countries in 2016. The STI Strategy showcases seven milestones for achievement by 2020 and four global targets for achievement by 2030 (Box 1) [3].

**Box 1. WHO STI strategy 2020 milestones and 2030 targets.**

**2020 WHO STI Strategy Milestones**:

- 70% of countries have sexually transmitted infection surveillance systems in place that are able to monitor progress towards the relevant targets
- 70% of countries have at least 95% of pregnant women screened for syphilis; and 95% of syphilis-seropositive pregnant women treated with at least one dose of intramuscular benzathine penicillin
- 70% of key populations for HIV have access to a full range of services relevant to sexually transmitted infection and HIV, including condoms
- 70% of countries provide sexually transmitted infection services or links to such services in all primary, HIV, reproductive health, family planning, and antenatal and postnatal care services
- 70% of countries deliver HPV vaccines through the national immunization programme
- 70% of countries report on antimicrobial resistance in *N*. *gonorrhoeae*
- 90% national coverage sustained and at least 80% in every district (or equivalent administrative unit) in countries with the human papillomavirus vaccine in their national immunization programme

**2030 WHO STI Strategy Targets**:

- 90% reduction of *T*. *pallidum* incidence globally (2018 global baseline);
- 90% reduction in *N*. *gonorrhoeae* incidence globally (2018 global baseline);
- 50 or fewer cases of congenital syphilis per 100 000 live births in 80% of countries;
- Sustain 90% national coverage and at least 80% in every district (or equivalent administrative unit) in countries with the human papillomavirus vaccine in their national immunization programme.

To evaluate and report on national implementation of recommendations in the STI Strategy for the 74th World Health Assembly in 2021, WHO performed a national survey. This survey aimed to assess national level implementation and prioritization of STI control programming and achievement of the 2020 milestones in the STI Strategy as a measure of progress towards achievement of the 2030 global targets.

## Methods

In August 2019, a survey instrument in the form of a questionnaire for country completion was jointly developed by experienced technical personnel at WHO Headquarters (HQ) and WHO regional offices. The survey was made available in five languages: English, French, Spanish, Russian, and Portuguese.

The survey sought to assess progress towards achievement of the following 2020 milestones within the STI Strategy: (1) number of countries with a STI surveillance system in place; (2) number of countries with a national policy for universal screening of pregnant women for syphilis as a first step in signifying countries’ intent to achieve the milestone of at least 95% of pregnant women attending antenatal care being screened for syphilis; (3) percentage of key populations to the HIV epidemics with access to STI and HIV services; (4) number of countries providing STI services or links to such services; (5) number of countries reporting on antimicrobial resistance (AMR) of *N*. *gonorrhoeae*; and (6) number of countries including human papillomavirus (HPV) vaccination among girls within national immunization schedules, as a proxy for delivery of this vaccine. The survey also assessed programme service delivery, including use and availability of diagnostics and medications, and captured technical assistance needs in STI programming and surveillance (S1 File).

During October 2019 to March 2020, national STI, HIV or reproductive health programme directors or managers, national sexual and reproductive health officers or directors, national programme officers for maternal and child health, national disease surveillance coordinators, national laboratory surveillance officers or managers, WHO country programme officers, or other UN agency HIV, STI or sexual and reproductive health officers (UNICEF, UNAIDS, UNFPA) completed the questionnaires. These staff were encouraged to liaise with programme managers with responsibility for HIV, reproductive health, immunization and commodities to gather the non-STI information needed to complete the survey. Three email reminders were sent to non-responding countries.

Regional office participants were made aware in the invitation to participate and on the survey cover page that results would be used to inform WHO and the World Health Assembly on progress towards globally recommended STI programme implementation (S1 File). Participation in the survey was voluntary and the intent to assess national-level efforts in the prevention and control of STI was included on the survey cover page. Written completion of the survey was considered consent to participate in this public health survey. Collection of these data for public health purposes complies with 45 CFR 46 subpart a-46104 and as described under 45 CFR 164.512(b)(i); "public health activities and purposes" as not human subjects’ research.

Completed questionnaires were returned through WHO regional offices to WHO HQ. Data were compiled and entered in Open Clinica^™^ [4] during February-May 2020 and analysed using SPSS (Chicago, IL, USA), SAS (Cary, NC), and Microsoft Excel (Redmond, WA, USA).

Descriptive analyses included frequency and percentages calculations for each survey question, using the number of completed responses as the denominator. Analyses were based on the overall responses received from reporting countries. Missing responses were not included in the denominator for percentage calculations. Analyses were reported for the six WHO regions and by World Bank income classifications [5]. Responses received from these reporting countries are considered a proxy for the performance of the 2020 STI Strategy milestones by WHO Member States.

## Results

Of the 194 WHO Member States to which the survey was distributed, 112 (58%) submitted a survey response (S1 Table). The regions with the highest survey response rates were South-East Asia (91%, 10/11), Americas (71%, 25/35,) and Western Pacific (67%, 18/27). The lowest survey completion rate was from the Eastern Mediterranean region (29%, 6/21). World Bank income classifications [5] of responding countries included high-income (27%, 30/112), upper-middle income (32%, 36/112), lower-middle income (24%, 27/112), and low income (17%, 19/112) (S2 Table).

### WHO STI strategy and member state national STI strategies

Ninety-two percent (103/112) of the reporting countries were familiar with the GHSS, 2016–2021 and 64% (72/112) had a national STI strategy, ranging from 37% (10/27) of countries in the European Region to 77% (20/26) of countries in the African Region (Tables 1 and 2). Forty-two countries updated their national strategies after the launch of the WHO global STI strategy (58%, 42/72). The WHO strategy was used as a reference in all 42 of these national strategy updates. In 84% (62/72) of reporting countries, the national STI strategy was integrated within the HIV national strategy.

**Table 1:** National policies and guidelines for STI surveillance and programming by WHO Region.

Region	National STI strategy	STI surveillance or monitoring system	Strategy for EMTCT*	STI treatment guidelines	AMR* surveillance for *N*. *gonorrhoeae*	HPV* vaccine in national immunization plan
**African**	20/26 (77%)	22/26 (85%)	19/26 (73%)	24/27 (92%)	11/19 (58%)	8/26 (31%)
**Americas**	16/25 (64%)	21/25 (84%)	22/25 (88%)	22/25 (88%)	11/19 (58%)	20/25 (80%)
**South-East Asia**	8/10 (80%)	10/10 (100%)	10/10 (100%)	10/10 (100%)	6/10 (60%)	4/10 (40%)
**European**	10/27 (37%)	25/27 (93%)	13/27 (48%)	19/27 (70%)	18/22 (82%)	20/26 (77%)
**Eastern Mediterranean**	4/6 (67%)	4/6 (67%)	5/6 (83%)	4/6 (67%)	2/4 (50%)	1/5 (20%)
**Western Pacific**	14/18 (78%)	15/17 (88%)	9/17 (50%)	17/18 (94%)	9/15 (60%)	12/18 (67%)
**Total**	72/112 (64%)	97/111 (87%)	78/111 (70%)	96/112 (86%)	57/89 (64%)	65/110 (59%)

**Table 2:** National policies and guidelines for STI surveillance and programming by World Bank income classification.

World Bank Income Classification	National STI strategy	STI surveillance or monitoring system	Strategy for EMTCT*	STI treatment guidelines	AMR* surveillance for *N*. *gonorrhoeae*	HPV* vaccine in national immunization plan
**High income**	12/30 (40%)	29/30 (97%)	16/29 (45%)	21/30 (70%)	24/27 (89%)	28/30 (93%)
**Upper middle income**	24/36 (67%)	30/36 (83%)	30/36 (83%)	32/36 (89%)	16/26 (62%)	23/36 (64%)
**Lower middle income**	20/27 (74%)	21/26 (81%)	21/27 (78%)	25/27 (93%)	9/22 (41%)	8/25 (32%)
**Low income**	16/19 (84%)	17/19 (90%)	14/19 (74%)	18/19 (95%)	8/14 (57%)	6/19 (32%)
**Total**	72/112 (64%)	97/111 (87%)	78/111 (70%)	96/112 (86%)	57/89 (64%)	65/110 (59%)

*Elimination of mother-to-child transmission of HIV and syphilis (EMTCT), anti-microbial resistance (AMR), human papillomavirus (HPV).

### Elimination of mother to child transmission of HIV, syphilis, and hepatitis B

Seventy-eight countries reported having a national strategy for the elimination of mother-to-child transmission (EMTCT) of HIV and syphilis (70%, 78/112) (Tables 1 and 2) and 58 (74%, 58/79) countries reported having a plan to apply for WHO validation of EMTCT. Of the nine STI interventions evaluated, EMTCT was ranked as a high priority by the greatest number of countries (Table 3). Although less than half (45%) of high income countries reporting having a national strategy for EMTCT (S3 Table), 93% (103/111) of countries reported having a national policy for screening all pregnant women for syphilis, 93% (104/108) for HIV, and 74% (84/110) for hepatitis B. For pregnant women, 96% (107/111) of countries reported routinely offering screening for syphilis and 94% (103/110) for HIV screening. These data suggest that, based on available policy and reported service delivery as first steps, the GHSS milestone of 95% coverage of HIV and syphilis screening in pregnant women is prioritized for achievement among these reporting countries.

**Table 3:** Country prioritization of STI interventions (N = 111).

Intervention	High	Medium	Low	Not a priority or not done
EMTCT*	100 (90%)	7 (6%)	4 (4%)	0
STI screening conducted among persons with HIV	86 (77%)	20 (18%)	3 (3%)	2 (2%)
STI screening among high-risk populations of MSM and SW	79 (71%)	16 (14%)	10 (9%)	6 (5%)
Condom distribution	74 (67%)	25 (23%)	9 (8%)	3 (3%)
HPV vaccine for young women	65 (59%)	14 (13%)	18 (16%)	13 (12%)
STI syndromic management	62 (56%)	26 (23%)	10 (9%)	13 (12%)
STI surveillance and monitoring	62 (56%)	30 (27%)	15 (14%)	4 (4%)
Provision of STI services for adolescents	50 (45%)	34 (31%)	22 (20%)	58 (5%)
Antimicrobial resistance monitoring of gonococcal isolates	38 (34%)	30 (27%)	24 (22%)	19 (17%)

* Elimination of mother-to-child transmission of HIV & syphilis.

### STI surveillance systems and monitoring for antimicrobial resistance in Neisseria gonorrhoeae isolates

Availability of STI surveillance was reported by 87% (97/110) of countries responding to this entry (Tables 1 and 2). Ninety-six countries (88%, 96/110) use STI case reporting as part of surveillance using syndromic and/or etiologic case diagnosis with some countries using a mixture of both. Countries reported variable performance of other specific WHO-recommended elements of national STI surveillance (S4 Table) [6]. The STI Strategy milestone of having 70% of countries with a STI surveillance system in place was achieved for this group of reporting countries.

Overall, 80% (86/108) of responding countries reported conducting antimicrobial susceptibility testing. Only 89 countries responded to the survey question related to gonococcal antimicrobial susceptibility testing. Of these 64% (57/89), primarily high-income countries, reported conducting this surveillance (Tables 1 and 2, S5 Table). These results do not indicate achievement of the 2020 STI Strategy milestone of 70% of countries performing surveillance for AMR for *N gonorrhoeae*. Only 56% (62/110) of responding countries reported overall STI surveillance to be a high priority STI intervention and only 34% (38/110) ranked surveillance for AMR in *N gonorrhoeae* to be of high priority (Table 3).

### Screening, diagnosis, and treatment of STIs

Of reporting countries, 88% (99/112) provide STI care at primary health care services, 91% (103/112) within HIV services, 85% (95/112) in reproductive health clinics, 77% (86/112) in family planning clinics, 89% (100/112) in antenatal/postnatal clinics. and 85% (95/112) in specialized STI clinics. These results suggest achievement of the STI Strategy milestone of 70% of countries providing STI services or links to such services in primary, HIV, reproductive health, family planning, and pre- and post-natal care services.

Countries reported the highest priority STI services as EMTCT of HIV and syphilis (90%, 100/111), STI screening among persons with HIV (77%, 86/111), STI screening among high-risk populations of men who have sex with men (MSM) and sex workers (SW) (71%, 79/111), condom distribution (71%, 74/111) and HPV vaccination for young women (59%, 65/111) (Table 3).

Availability of HIV and syphilis testing was reported by the highest proportion of countries (95%, 105/111) while the proportion of countries reporting the availability of chlamydia, HPV, and herpes testing was lower at 59% (66/111), 56% (62/111) and 54% (60/111), respectively.

For pregnant women, screening was routinely offered by 94% (103/110) of countries for HIV, 96% (107/111) for syphilis, 74% (81/110) for hepatitis B, 28% (31/110) for hepatitis C, 27% (30/110) for gonorrhoea, 20% (22/110) for chlamydia, 17% (19/110) for HPV, and 15% (17/110) for herpes.

For key populations, 83% (93/112) of countries reported providing HIV screening to MSM, 74% (83/112) for syphilis, 50% (56/112) for gonorrhoea, 41% for chlamydia (46/111) and 54% (60/111) for hepatitis B. For SW, 81% (90/111) of countries reported providing screening for HIV, 73% (81/111) for syphilis, 49% (54/110) for gonorrhoea, 44% (48/110) for chlamydia and 49% (54/110) for hepatitis B. These results describe screening services available towards the milestone achievement of 70% of key populations having access to a full range of services relevant to sexually transmitted infections and HIV.

Recommended treatment for syndromic management of urethral discharge in males included azithromycin in 85% (91/107) of reporting countries and ceftriaxone in 83% (89/107). Recommended treatment for vaginal discharge included azithromycin in 76% (80/106) and ceftriaxone in 73% (77/105) of reporting countries. For presumptive treatment of genital ulcer disease syndrome, benzathine penicillin was used by 89% (93/105) and acyclovir by 78% (82/105) of reporting countries.

STI medication stock outs during 2015–2019 were reported by 34% (37/110) of countries. The most frequent medication stock out was benzathine penicillin, reported by 34% of countries (37/110) followed by ceftriaxone 15% (17/111), acyclovir 14% (16/111), and cefixime 14% (15/110). Recent stock outs of any STI medication were reported by 10 countries in 2018 and by seven countries in 2019. Stock outs of syphilis test kits were reported by 57% (30/53) of responding countries.

### Human papillomavirus (HPV) and cervical cancer

The HPV vaccine was reported to be included in the national immunization schedule of 59% (65/110) of reporting countries (Tables 1 and 2) and was considered as high priority by 59% of countries (65/111) (Table 3). Only 32% of low- and low-middle income countries included HPV in their national immunization schedule (Table 2). Country responses did not meet the 2020 STI Strategy milestone of 70% of countries delivering HPV vaccines through the national immunization programme.

Among the 65 countries that include the HPV vaccine in the national immunization schedule, the vaccine is specifically recommended for adolescent girls in 94% (63/65) and is recommended for both adolescent girls and boys in 31% (20/111). The HPV vaccine is recommended for persons living with HIV in 28% (18/65) and for MSM in 18% (12/65) of reporting countries. Of the countries with a vaccination program for boys (along with girls, n = 20), only 7 (35%) also include HPV vaccination for MSM. Populations included for HPV vaccination varied by World Bank income classification (S6 Table).

Of reporting countries, 94% (102/109) reported availability of cervical cancer screening for general populations of women between the ages of 35 and 55. Among these countries, the tests in use included PAP smear in 91% (95/104), HPV test 48% (49/102), and visual inspection with acetic acid testing 53% (53/100).

Methods of treatment of cervical pre-cancerous and cancerous lesions reported by countries included: loop electrosurgical excision procedure (79%, 81/102), surgical removal (75%, 77/102), cryotherapy (72%, 73/102), and thermal ablation (47%, 47/101).

### Request for and sources of technical assistance

WHO technical assistance was requested by 75% (82/109) of countries. The areas of technical assistance need included: development of national strategy (76%, 62/82), STI treatment (59%, 48/81), STI surveillance (78%, 63/81), EMTCT (66%, 55/83), AMR of gonorrhoea (78%, 63/81), and HPV prevention and treatment (68%, 56/82). Countries reported the main technical partners or agencies that supported STI prevention and control services to include the US Centers for Disease Control and Prevention, European Center for Disease Control (ECDC), the Global Fund, WHO, UNAIDS and other UN agencies (UNFPA, UNICEF, UNDP). The main sources of funding support for implementation of STI-related prevention and control programmes were reported as national governments and the Global Fund.

## Discussion

The STI Strategy urges countries to define and implement national strategies to prevent and control STIs, informed by national STI surveillance and programme data. Among the 112 countries that responded to the survey, only 64% reported having a national STI strategy. EMTCT of HIV and/or syphilis emerged as a common goal based on availability of national strategies, prioritization, and service delivery. A higher percentage of responding countries reported conducting STI surveillance or monitoring (87%), demonstrating the opportunity for development of national STI strategies guided by national STI monitoring and STI service delivery. A majority (70%) of countries reported providing STI services or links to STI services in primary care, HIV, reproductive health, family planning, and pre- and post-natal care services and for key populations. Medication and test kits stock outs, primarily of benzathine penicillin and syphilis test kits suggest that efforts to control adult, maternal and congenital syphilis may have been compromised in recent years. HPV vaccination recommendations, cervical cancer screening, and treatment of cervical lesions indicate opportunities for improved service delivery. Delivery of these and other STI services varied by country income classification. Guided by surveillance, concerted efforts to expand and improve the delivery of STI clinical services for general and key populations are needed to reach the 2030 targets of the Global STI Strategy. Direct technical assistance by WHO was requested by over 70% of surveyed countries and will be needed to accelerate progress in these areas.

As of December 2021, WHO has validated 15 countries as having achieved elimination of mother-to-child transmission of HIV and/or syphilis [7] and has integrated hepatitis B into the global EMTCT framework. The proportions of responding countries with national strategies for EMTCT (70%) and those reporting offering HIV and syphilis (90%) and hepatitis B screening for pregnant women (74%) were high. Improved service delivery and surveillance for congenital syphilis prevention and national-level incorporation of hepatitis B into EMTCT strategies are key targets to reduce gaps in antenatal and infant care service delivery and elevate these prevention interventions to the levels achieved for HIV [8, 9]. The global STI strategy calls for reduction of mother-to-child transmission of syphilis (congenital syphilis) to the elimination threshold of 50 cases/100,000 live births by 2030 [3]. Efforts to secure and administer syphilis testing and treatment commodities will be critical to this achievement.

Although a majority of countries reported conducting STI surveillance, in most settings surveillance primarily reflects case reporting. In countries with limited laboratory-based diagnostic testing, case reports will reflect syndromic diagnosis of the common STI clinical presentations of urethral and vaginal discharge and genital ulcer disease [10]. Due to the large proportion of STIs that present without symptoms [11], basing national STI burden estimates on syndromic case reports alone provides insufficient data to guide national programming. Few countries reported conducting syndromic or etiologic prevalence surveys. Although expensive, etiologic surveys of syndromes, sentinel surveillance and general population STI prevalence surveys can be used to enhance and improve national STI surveillance and estimation to better guide programming, prioritization and resource mobilization. Performance of gonococcal AMR surveillance was reported by just over half of reporting countries. This is consistent with prior reports but demonstrates limited expansion [12]. WHO has STI surveillance tools that can be utilized to generate national estimates of STIs and to guide antimicrobial resistance monitoring for *N*. *gonorrhoeae* [13, 14]. WHO also offers technical assistance and training in performing national STI surveillance assessments and estimates using published tools [6, 15–19].

A majority of countries reported the availability of STI clinical services or links within other clinical settings such as primary care, HIV, reproductive health, family planning, and pre- and post-natal care services. STI screening among key populations of MSM and SW as well as pregnant women was primarily focused on HIV and syphilis with fewer countries reporting hepatitis B, chlamydia, and gonorrhoea screening. Quality STI services rely on appropriate diagnosis and treatment. Availability of diagnostic testing for the general population was reported by 96% of countries for HIV and syphilis but far fewer countries in comparison reported available testing for chlamydia, gonorrhoea, HPV, and herpes. These findings reflect the need for expanded availability of STI diagnostics and transition from syndromic management to etiological testing to improve diagnostic accuracy and efficacy of treatment. Although STI services may be available at multiple access points, the quality of these services may be compromised if etiologic testing is not available and treatment is not based on updated syndromic management guidelines [20, 21].

Cervical cancer is the fourth leading cancer among women globally, claiming the lives of over 300,000 annually [22]. In August 2020, the World Health Assembly adopted a global strategy to accelerate cervical cancer elimination [23]. The first pillar of this strategy calls for 90% of young women to receive the HPV vaccine. The global STI strategy milestone of 70% of countries having the HPV vaccine in national immunization schedules was not demonstrated by this group of 112 reporting countries (59%), suggesting the need for advocacy, political support and funding for countries to adopt this critical vaccine programming. Cervical cancer screening is the second pillar of the global elimination strategy and was reported as available using multiple modes of testing by 94% of reporting countries. Finally, related to the third pillar of the cervical cancer elimination strategy [23], reporting of treatment of cancerous and pre-cancerous cervical lesions by treatment modality was available from 102 countries. These findings can provide a baseline for monitoring country adoption of these elements of the global cervical cancer elimination strategy.

The response rate of 58% (112/194) of surveyed WHO Member States should be considered when interpreting these data. This analysis was conducted using number of reporting countries as the denominator. Countries that did not report (particularly low-middle- and low- income countries) may not have comparable implementation. The proportion of responding countries from high, and upper-middle income countries (59%, 66/112), and low-middle- and low- income countries (41%, 46/112) was equal to the overall proportion of WHO Member States within these World Bank income designations of 59%, (115/194) and 41% (79/194) respectively. Nonetheless, these survey results may over or underestimate implementation of STI response activities at country, regional, and global levels. We specifically note the limited representation of two specific groups of countries: (1) only one of nine Portuguese-speaking countries completed the survey despite translation and outreach to these countries by a Portuguese-speaking staff member and (2) few (29%, 6/21) countries in the Eastern Mediterranean Region completed the survey. We did not include individual country responses, instead grouping responses by World Bank income classification and WHO region. For some surveillance activities such as AMR monitoring for *N*. *gonorrhoeae*, a greater number of high- and high-middle income countries are known to perform this surveillance [9]. Other global monitoring systems may reflect different levels of performance from those reported here. We used policy indicators as a marker for service delivery to assess some milestones, which assumes the policies are universally implemented in clinical care settings. However, the availability of policies is not likely to adequately reflect the level and quality of service delivery. Country-level coverage of syphilis screening and treatment among pregnant women was not assessed through this survey as these are reported by countries separately through the Global AIDS Monitoring System [24]. Similarly, the milestones of achievement of 70% of HIV key populations having access to services and 90% HPV vaccine coverage among target populations could not specifically be evaluated by this survey. Although we have reported on the achievement of the 2020 milestones in the STI strategy by these respondent countries as a proxy for all WHO Member States, generalization of these findings globally should consider the response rate and regions with limited representation.

In 2019, an interim assessment of progress towards achieving the milestones for the three interlinked global strategies for HIV, hepatitis and STIs concluded that limited progress on STI prevention and elimination had been made compared to progress for HIV and hepatitis [25]. Results from this survey identified specific programme areas for improvement including national STI strategy development; etiologic case and prevalence surveillance; screening, diagnosis and treatment of STIs; and cervical cancer prevention and treatment. These findings are being used to inform the development of the WHO Health Sector Strategy on STI for 2022–2030 [26] considering the challenges and opportunities that exist within current health care systems [27]. Monitoring country progress towards achievement of the milestones of the global STI strategy serves to provide strategic information and highlight gaps and opportunities for prioritization and investment by countries, global stakeholders and international donors. Guided by the information provided by countries in the survey, WHO will continue to support countries by providing guidance and technical assistance for the development and improvement of programming for STI service delivery and surveillance.

## Supporting information

S1 File Evaluation of the global STI strategy: Country STI survey STI activities assessment at country level. (PDF)

S1 Table Countries responding to the WHO STI global health sector strategy survey (N = 112). (DOCX)

S2 Table Survey response rates by WHO region and World Bank income classification. (DOCX)

S3 Table National EMTCT* policy and programming by World Bank income classification. * Elimination of mother-to-child transmission of HIV and syphilis. **16 between 2020–2023.
(DOCX)

S4 Table National STI surveillance system elements by World Bank income classification. (DOCX)

S5 Table Laboratory antimicrobial resistance (AMR) surveillance by World Bank income classification. (DOCX)

S6 Table National human papillomavirus virus (HPV) vaccine schedules by World Bank income classification. (DOCX)

S1 Checklist *PLOS ONE* clinical studies checklist. (DOCX)
