# Supplementary material for: Assessment of country implementation of the WHO global health sector strategy on sexually transmitted infections (2016-2021)
Source: PLoS One. 2022 May 4;17(5):e0263550. doi: 10.1371/journal.pone.0263550 (PMC9067912; doi:10.1371/journal.pone.0263550)
Supplement: S1 File — (PDF) [file pone.0263550.s001.pdf]

World Health Organization	Evaluation of the Global STI Strategy: Country STI Survey<br>STI Activities Assessment at Country Level	CPG<br>Page 1/1<br>V1.0 (11 Jun 2019)

PROJECT ID:

A	6	5	9	9	6

Date form completed:

Day		Month		Year			
				2	0	1	9

This questionnaire will take approximately 30 minutes to complete. Thank you for taking the time.

This information is essential for the assessment of interventions and activities that have taken place since the launching of the Global Strategy on Sexually Transmitted Infections (STI). The information will not only assist WHO in compiling its report to the World Health Assembly, but also countries themselves to understand what has been happening around the prevention and control of STI. The questionnaire will give a high level assessment of STI prevention and control efforts, and identify areas in need of strengthening to achieve maximum coverage of STI prevention and care activities.

1. Name of Country: ______________________________

2. Name of Person or Persons Completing this Form: ______________________________

3. Job titles/description: ☐

   1= National STI programme director or manager
   2= National HIV programme director or manager
   3= National sexual and reproductive health officer or director
   4= National programme officer for maternal and child health
   5= National disease surveillance coordinator
   6= National laboratory surveillance officer or manager
   7= WHO country programme officer
   8= Other UN agency HIV, STI or sexual and reproductive health officer (UNICEF, UNAIDS, UNFPA)
   9= Other

   3a) If ***Other*** , please specify: ______________________________

4. Address: ______________________________

____

5. Telephone number: ______________________________

6. Mobile number: ______________________________

7. E-mail address: ______________________________

To be completed by Responder (the responder will consolidate the information if more than one person is involved in the process):

Name: ______________________________

Signature: ______________________________

Date:

Day		Month		Year			
				2	0	1	9

PROJECT ID: A 6 5 9 9 6

Date form completed:

Day		Month		Year			
				2	0	1	9

Name of Country: ____________________

I - STI strategy

1. Are you familiar with (have a copy or have the link to) the WHO Global Health Sector Strategy on Sexually Transmitted Infections, 2016-2021? ☐
   1= Yes 2= No

2. Does the country have an official National STI Strategy? ☐
   1= Yes 2= No (***Go to*** Q3)

   2a) When was it last updated? (year) ☐☐☐☐
   9999 if ***Don't know*** or ***Data not available***

   2b) Please provide the WEB link of National STI Strategy:
   2b1) Provide WEB Link if ***Available*** : ____________________
   OR
   2b2) Check the box if WEB link is ***Not available:*** ☐

   2c) Is the National STI Strategy/Programme separate from the National HIV Strategy/Programme? ☐
   1= Yes, separate
   2= No, integrated with HIV strategy
   3= Do not have an STI programme

   2d) Was the WHO Global Health Sector Strategy on Sexually Transmitted Infections 2016 used as a reference to develop or to update the National STI Strategy or Programme? ☐
   1= Yes 2= No 3= Don't know 4= Data not available

   2e) Has your National STI Strategy/Programme been disseminated and how? ☐
   1= Yes 2= No, not formally distributed (***Go to*** Q3)

   2e1) Posted on website: ☐
   1= Yes 2= No (***Go to*** Q2e2)
   2e1s) If ***Yes*** , please provide the link: ____________________

   2e2) Email distribution: ☐
   1= Yes 2= No (***Go to*** Q2e3)
   If ***Yes*** , to whom/at what level:

	Yes	No
2e2a) Sub-national (provinces/states)	☐	☐
2e2b) Districts	☐	☐
2e2c) Clinics	☐	☐
2e2d) Clinicians/care providers	☐	☐
2e2e) NGOs/other organizations	☐	☐

   2e3) Hard copies distributed: ☐
   1= Yes 2= No (***Go to*** Q2e4)
   If ***Yes*** , to whom/at what level:

	Yes	No
2e3a) Sub-national (provinces/states)	☐	☐
2e3b) Districts	☐	☐
2e3c) Clinics	☐	☐
2e3d) Clinicians/care providers	☐	☐
2e3e) NGOs/other organizations	☐	☐

PROJECT ID: A 6 5 9 9 6

Date form completed:

Day		Month		Year			
				2	0	1	9

Name of Country: ______________________

I - STI strategy (*Continued*)

2e4) Trainings/oral presentations or other forms of face-to-face meetings: ☐
1= Yes 2= No (***Go to*** Q2e5)
If ***Yes***, to whom/at what level:

	Yes	No
2e4a) Sub-national (provinces/states)	☐	☐
2e4b) Districts	☐	☐
2e4c) Clinics	☐	☐
2e4d) Clinicians/care providers	☐	☐
2e4e) NGOs/other organizations	☐	☐

2e5) Other: ☐
1= Yes 2= No (***Go to*** Q3)
2e5s) If Other= ***Yes***, please provide a short description of the process: ______________________

____

3. Has the WHO Global Health Sector Strategy on Sexually Transmitted Infections 2016 been used to guide any new or updated STI policies or programmatic activities? ☐
1= Yes 2= No 3= Don't know 4= Data not available

4. Has the WHO Global Health Sector Strategy on Sexually Transmitted Infections 2016 been disseminated within your country and how? ☐
1= Yes 2= No, not formally distributed (***Go to*** Q5)

4a) Posted on website: ☐
1= Yes 2= No (***Go to*** Q4b)
4as) If ***Yes***, please provide the link: ______________________

4b) Email distribution: ☐
1= Yes 2= No (***Go to*** Q4c)
If ***Yes***, to whom/at what level:

	Yes	No
4b1) Sub-national (provinces/states)	☐	☐
4b2) Districts	☐	☐
4b3) Clinics	☐	☐
4b4) Clinicians/care providers	☐	☐
4b5) NGOs/other organizations	☐	☐

4c) Hard copies distributed: ☐
1= Yes 2= No (***Go to*** Q4d)
If ***Yes***, to whom/at what level:

	Yes	No
4c1) Sub-national (provinces/states)	☐	☐
4c2) Districts	☐	☐
4c3) Clinics	☐	☐
4c4) Clinicians/care providers	☐	☐
4c5) NGOs/other organizations	☐	☐

PROJECT ID:

A	6	5	9	9	6

Date form completed:

Day		Month		Year			
				2	0	1	9

Name of Country: ______________________

I - STI strategy (*Continued*)

4d) Trainings/oral presentations or other forms of face-to-face meetings: ☐
1= Yes 2= No (**Go to** Q4e)
If ***Yes***, to whom/at what level:

	Yes	No
4d1) Sub-national (provinces/states)	☐	☐
4d2) Districts	☐	☐
4d3) Clinics	☐	☐
4d4) Clinicians/care providers	☐	☐
4d5) NGOs/other organizations	☐	☐

4e) Other: ☐
1= Yes 2= No (**Go to** Q5)
4es) If Other= ***Yes***, please provide a short description of the process: ______________________

____

II - STI treatment guidelines

5. Are you familiar with (have copies or have the links to) the 2016 WHO STI Treatment Guidelines?

	Yes	No
5a) Neisseria gonorrhoeae (gonorrhoea)	☐	☐
5b) Chlamydia trachomatis (chlamydia)	☐	☐
5c) Herpes simplex virus: (Genital herpes)	☐	☐
5d) Treponema pallidum (syphilis)	☐	☐

6. Does the country have formal national STI treatment guidelines? ☐
1= Yes 2= No (**Go to** Q6d)

If Q6= Yes, Complete Q6a, Q6b, Q6c and Go to Q7.
If Q6= No, Complete Q6d and Go to Q7.

6a) If ***Q6= Yes***, when was it last updated? (year) ☐☐☐☐
9999 if ***Don't know*** or ***Data not available***

6b) If ***Q6= Yes***, were the 2016 WHO STI treatment guidelines used as a reference for the development or update of the national STI guidelines? ☐
1= Yes 2= No 3= Don't know 4= Data not available

6c) If ***Q6= Yes***, specify which reference documents have been used to develop national STI treatment guidelines?

	Yes	No
6c1) WHO STI treatment guidelines	☐	☐
6c2) US CDC STD treatment guidelines	☐	☐
6c3) STI treatment guidelines from a neighbour country	☐	☐
6c4) International Union of STI treatment guidelines	☐	☐
6c5) BASHH Guidelines - British Association for Sexual Health and HIV	☐	☐
6c6) Other	☐	☐

6c6s) If ***Other= Yes***, please specify: ______________________

PROJECT ID: A 6 5 9 9 6

Date form completed:

Day		Month		Year			
				2	0	1	9

Name of Country: ______________________

II - STI treatment guidelines (*Continued*)

6d) If ***Q6= No***, specify which documents are used to guide treatment of STIs?

	Yes	No
6d1) WHO STI treatment guidelines	☐	☐
6d2) US CDC STD treatment guidelines	☐	☐
6d3) STI treatment guidelines from a neighbour country	☐	☐
6d4) International Union of STI treatment guidelines	☐	☐
6d5) BASHH Guidelines - British Association for Sexual Health and HIV	☐	☐
6d6) Other	☐	☐

6d6s) If ***Other= Yes***, please specify: ______________________

7. Have any STI treatment guidelines been disseminated in your country and how? ☐
1= Yes 2= No, not formally distributed (***Go to*** Q8)

7a) Posted on website: 1= Yes 2= No (***Go to*** Q7b) ☐
7as) If ***Yes***, please provide the link: ______________________

7b) Email distribution: 1= Yes 2= No (***Go to*** Q7c) ☐
If ***Yes***, to whom/at what level:

	Yes	No
7b1) Sub-national (provinces/states)	☐	☐
7b2) Districts	☐	☐
7b3) Clinics	☐	☐
7b4) Clinicians/care providers	☐	☐
7b5) NGOs/other organizations	☐	☐

7c) Hard copies distributed: 1= Yes 2= No (***Go to*** Q7d) ☐
If ***Yes***, to whom/at what level:

	Yes	No
7c1) Sub-national (provinces/states)	☐	☐
7c2) Districts	☐	☐
7c3) Clinics	☐	☐
7c4) Clinicians/care providers	☐	☐
7c5) NGOs/other organizations	☐	☐

7d) Trainings/oral presentations or other forms of face-to-face meetings: ☐
1= Yes 2= No (***Go to*** Q7e)
If ***Yes***, to whom/at what level:

	Yes	No
7d1) Sub-national (provinces/states)	☐	☐
7d2) Districts	☐	☐
7d3) Clinics	☐	☐
7d4) Clinicians/care providers	☐	☐
7d5) NGOs/other organizations	☐	☐

7e) Other: 1= Yes 2= No (***Go to*** Q8) ☐
7es) If ***Other= Yes***, please provide a short description of the process: ______________________

____

PROJECT ID: A 6 5 9 9 6

Date form completed:

Day		Month		Year			
				2	0	1	9

Name of Country: ______________________

III - National STI priorities

8. Please designate the priority level of each of these interventions in your country for STI control and prevention.
Choose one priority level for programme implementation (High, Medium, Low, Not a Priority or Not Done) per intervention:

INTERVENTION	**HIGH**	**MEDIUM**	**LOW**	**NOT A PRIORITY OR NOT DONE**
8a) Elimination of mother-to-child Transmission of HIV & syphilis	☐	☐	☐	☐
8b) HPV Vaccine for young women	☐	☐	☐	☐
8c) Condom distribution	☐	☐	☐	☐
8d) STI syndromic management	☐	☐	☐	☐
8e) STI surveillance and monitoring	☐	☐	☐	☐
8f) Antimicrobial resistance monitoring of gonococcal isolates	☐	☐	☐	☐
8g) STI screening among high-risk populations of MSM and sex workers	☐	☐	☐	☐
8h) STI screening conducted among persons with HIV	☐	☐	☐	☐
8i) Provision of STI services for adolescents	☐	☐	☐	☐

IV - Location of STI care services

9. Sexually transmitted infection care services are provided in which of the following clinical settings?

Note: Presence of STI care services is defined as presence of a health professional capable of STI management, including diagnosis (syndromic or etiological), treatment, counseling, and partner management

	**Yes**	**No**	If ***Yes*** , estimated percentage of all STI care delivered in this setting<br>999 if ***Don't know*** or ***Data not available***
9a) Primary care clinics	☐	☐	☐☐☐ %
9b) Reproductive health clinics	☐	☐	☐☐☐ %
9c) HIV clinics	☐	☐	☐☐☐ %
9d) Family planning clinics	☐	☐	☐☐☐ %
9e) Antenatal/postnatal care clinics	☐	☐	☐☐☐ %
9f) Specialized STI clinics	☐	☐	☐☐☐ %
9g) Key population services (MSM, sex workers)	☐	☐	☐☐☐ %
9h) Adolescent healthcare services	☐	☐	☐☐☐ %
9i) Prison settings	☐	☐	☐☐☐ %

PROJECT ID: A 6 5 9 9 6

Date form completed:

Day	Month	Year
		2 0 1 9

Name of Country: ____________________

V - Surveillance systems

10. Is STI surveillance or monitoring being done in your country? ☐
1= Yes 2= No ***(Go to*** **Q11)**

10a) If ***Yes*** , Which surveillance or monitoring elements are included?

	Yes	No
10a1) Periodic STI prevalence survey among general and high risk populations	☐	☐
10a2) Etiologic assessment of STI syndromes	☐	☐
10a3) STI case reporting	☐	☐

10b) If ***Q10a3= Yes*** , which STIs are reported at different levels of the health system?

	a- Universal		b- STI Clinics		c- Sentinel Sites		d- Private Sector		e- Laboratories	
	Yes	No	Yes	No	Yes	No	Yes	No	Yes	No
10b1) Syndrome: Urethral discharge	☐	☐	☐	☐	☐	☐	☐	☐		
10b2) Syndrome: Genital Ulcer Disease	☐	☐	☐	☐	☐	☐	☐	☐		
10b3) Syndrome: Vaginal discharge	☐	☐	☐	☐	☐	☐	☐	☐		
10b4) Etiologies: Syphilis	☐	☐	☐	☐	☐	☐	☐	☐	☐	☐
10b5) Etiologies: Gonorrhoea	☐	☐	☐	☐	☐	☐	☐	☐	☐	☐
10b6) Etiologies: Chlamydia	☐	☐	☐	☐	☐	☐	☐	☐	☐	☐
10b7) Etiologies: Trichomoniasis	☐	☐	☐	☐	☐	☐	☐	☐	☐	☐
10b8) Etiologies: Syphilis in pregnant women	☐	☐	☐	☐	☐	☐	☐	☐	☐	☐

10c) Is STI surveillance or monitoring integrated within the national health information system (NHIS)? ☐
1= Yes 2= No

10d) Is the surveillance data disaggregated by:

	Yes	No
10d1) age:	☐	☐
10d2) sex:	☐	☐
10d3) location:	☐	☐
10d4) population:	☐	☐

11. Is the country entering HIV indicators into the UNAIDS Global AIDS Monitoring System? ☐
1= Yes **2= No (Go to Q13)**

PROJECT ID: A 6 5 9 9 6

Date form completed:

Day		Month		Year			
				2	0	1	9

Name of Country: ____________________

V - Surveillance systems (*Continued*)

12. Is the country entering STI indicators into the UNAIDS Global AIDS Monitoring System? ☐
1= Yes (***Complete*** Q12a to Q12h and ***Go to*** Q13) 2= No (***Go to*** Q12i)

If **Q12= Yes**, check which STI indicators are entered into the UNAIDS Global AIDS Monitoring System:

	Yes	No
12a) Syphilis screening coverage among pregnant women in antenatal care clinics	☐	☐
12b) Syphilis positivity among pregnant women in antenatal care clinics	☐	☐
12c) Syphilis treatment coverage among pregnant women testing positive in antenatal care clinics	☐	☐
12d) Congenital syphilis case rate	☐	☐
12e) Syphilis screening and positivity in MSM	☐	☐
12f) Syphilis screening and positivity in sex workers	☐	☐
12g) Urethral discharge case rate in men	☐	☐
12h) Gonorrhoea case rate in men	☐	☐

12i) If ***Q12= No*** , why? ____________________

VI - Elimination of Mother to Child Transmission of HIV and Syphilis

13. Is there a national strategy for the Elimination of Mother-to-Child Transmission of HIV and Syphilis? ☐
1= Yes 2= No (***Go to*** Q14) 3= Yes, but for HIV only (***Go to*** Q14)

13a) If ***Yes*** , add and/or provide link to the CS case definition ____________________

13b) If ***Yes,*** is the country planning to validate elimination of vertical transmission of both HIV and syphilis or Path to Elimination?
1= Yes 2= No

13c) If ***Yes,*** which year is country planning to apply for WHO validation of elimination (EMTCT) or Path to Elimination? 2 0 ☐☐
99 if ***Don't know*** or ***Data not available***

14. Is there a national policy for screening pregnant women for HIV? ☐
1= Yes 2= No (***Go to*** Q15)

14a) If ***Yes*** , how many times during the pregnancy? ☐

14b) If ***Yes,*** is the country screening at delivery?
1= Yes 2= No

15. Is there a national policy for screening pregnant women for syphilis? ☐
1= Yes 2= No

15a) If ***Yes*** , how many times during the pregnancy? ☐

15b) If ***Yes,*** is the country screening at delivery?
1= Yes 2= No

PROJECT ID: A 6 5 9 9 6

Date form completed:

Day		Month		Year			
				2	0	1	9

Name of Country: ____________________

VI - Elimination of Mother to Child Transmission of HIV and Syphilis *(Continued)*

16. Are pregnant women routinely screened for any of the following STIs?

	Yes	No
16a) HIV	☐	☐
16b) Syphilis	☐	☐
16c) Gonorrhoea	☐	☐
16d) Chlamydia	☐	☐
16e) Trichomoniasis	☐	☐
16f) Human papillomavirus (HPV)	☐	☐
16g) Genital herpes	☐	☐
16h) Hepatitis B	☐	☐
16i) Hepatitis C	☐	☐
16h) Other	☐	☐

16hs) If ***Other= Yes*** , please specify: ____________________

17. Are rapid syphilis tests used in antenatal care (ANC) clinics? ☐
1= Yes 2= No (***Go to*** Q18)

17a) If your country uses rapid syphilis tests in ANC have you experienced stockouts of test kits? ☐
1= Yes 2= No

18. Are dual HIV/syphilis tests used in antenatal care (ANC) clinics? ☐
1= Yes 2= No (***Go to*** Q19)

18a) If ***Yes,*** please specify brand: ____________________

18b) If ***Yes,*** please specify price: ____________

VII - Screening, Diagnosis, and Treatment of STIs

19. Are key populations of men who have sex with men (MSM) and sex workers screened for any of the following STIs?

	1- MSM		2- Sex workers	
	Yes	No	Yes	No
19a) HIV	☐	☐	☐	☐
19b) Syphilis	☐	☐	☐	☐
19c) Gonorrhoea	☐	☐	☐	☐
19d) Chlamydia	☐	☐	☐	☐
19e) Trichomoniasis	☐	☐	☐	☐
19f) Human papillomavirus (HPV)	☐	☐	☐	☐
19g) Genital herpes	☐	☐	☐	☐
19h) Hepatitis B	☐	☐	☐	☐
19i) Hepatitis C	☐	☐	☐	☐
19j) Other	☐	☐	☐	☐

19js) If ***Other= Yes*** , please specify: ____________________

PROJECT ID:

A	6	5	9	9	6

Date form completed:

Day		Month			Year			
					2	0	1	9

Name of Country: ____________________

VII - Screening, Diagnosis, and Treatment of STIs *(Continued)*

20. Which of the following diagnostic tests are available for STI diagnosis for the general population?

	Yes	No
20a) HIV	☐	☐
20b) Syphilis	☐	☐
20c) Gonorrhoea	☐	☐
20d) Chlamydia	☐	☐
20e) Trichomoniasis	☐	☐
20f) Human papillomavirus (HPV)	☐	☐
20g) Genital herpes	☐	☐
20h) Hepatitis B	☐	☐
20i) Hepatitis C	☐	☐
20j) Other	☐	☐

20js) If ***Other= Yes*** , please specify: ____________________

21. Have any studies been conducted in the country to identify the common etiologies of STI syndromes (urethral discharge, vaginal discharge, genital ulcer disesase)? ☐
1= Yes 2= No (***Go to*** Q22)

21a) Year of the latest version of the study: ☐☐☐☐
9999 if ***Don't know*** or ***Data not available***

21b1) Provide WEB Link if ***Available*** : ____________________
OR
21b2) Check the box if WEB link is ***Not available:*** ☐

22. What are the recommended medications used for urethral discharge?

	Yes	No
22a) Acyclovir	☐	☐
22b) Azithromycin	☐	☐
22c) Benzathine penicillin	☐	☐
22d) Ceftriaxone	☐	☐
22e) Cefixime	☐	☐
22f) Doxycycline	☐	☐
22g) Metronidazole	☐	☐
22h) Other	☐	☐

22hs) If ***Other= Yes*** , please specify: ____________________

PROJECT ID:

A	6	5	9	9	6

Date form completed:

Day		Month		Year			
				2	0	1	9

Name of Country: ______________________________

VII - Screening, Diagnosis, and Treatment of STIs (*Continued*)

23. What are the recommended medications used for vaginal discharge?

	Yes	No
23a) Acyclovir	☐	☐
23b) Azithromycin	☐	☐
23c) Benzathine penicillin	☐	☐
23d) Ceftriaxone	☐	☐
23e) Cefixime	☐	☐
23f) Doxycycline	☐	☐
23g) Metronidazole	☐	☐
23h) Other	☐	☐

23hs) If ***Other= Yes*** , please specify: ______________________________

24. What are the recommended medications used for genital ulcer disease?

	Yes	No
24a) Acyclovir	☐	☐
24b) Azithromycin	☐	☐
24c) Benzathine penicillin	☐	☐
24d) Ceftriaxone	☐	☐
24e) Cefixime	☐	☐
24f) Doxycycline	☐	☐
24g) Metronidazole	☐	☐
24h) Other	☐	☐

24hs) If ***Other= Yes*** , please specify: ______________________________

25. Which medications are on the national formulary or procurement list for treatment of STIs?

	Yes	No
25a) Acyclovir	☐	☐
25b) Azithromycin	☐	☐
25c) Benzathine penicillin	☐	☐
25d) Ceftriaxone	☐	☐
25e) Cefixime	☐	☐
25f) Doxycycline	☐	☐
25g) Metronidazole	☐	☐
25h) Other	☐	☐

25hs) If ***Other= Yes*** , please specify: ______________________________

PROJECT ID: A 6 5 9 9 6

Date form completed:

Day	Month	Year
		2 0 1 9

Name of Country: ____________________

VII - Screening, Diagnosis, and Treatment of STIs (*Continued*)

26. Has the country experienced stockouts of STI medications?

If ***Yes***, year when stockout was identified
(9999 if ***Don't know*** or ***Data not available***)

	Yes	No	First identified	Last identified
26a) Acyclovir	☐	☐	☐☐☐☐	☐☐☐☐
26b) Azithromycin	☐	☐	☐☐☐☐	☐☐☐☐
26c) Benzathine penicillin	☐	☐	☐☐☐☐	☐☐☐☐
26d) Ceftriaxone	☐	☐	☐☐☐☐	☐☐☐☐
26e) Cefixime	☐	☐	☐☐☐☐	☐☐☐☐
26f) Doxycycline	☐	☐	☐☐☐☐	☐☐☐☐
26g) Metronidazole	☐	☐	☐☐☐☐	☐☐☐☐
26h) Crystalline penicillin	☐	☐	☐☐☐☐	☐☐☐☐
26i) Other	☐	☐	☐☐☐☐	☐☐☐☐

26is) If ***Other= Yes***, please specify: ____________________

VIII - Surveillance for Antimicrobial Resistance (AMR) in Gonorrhoea/Gonococcal isolates

27. Is antimicrobial susceptibility testing performed by your national reference lab? ☐
1= Yes 2= No (***Go to*** Q29)

27a) Is antimicrobial susceptibility testing for gonorrhoea performed by your national reference lab? ☐
1= Yes 2= No (***Go to*** Q29)
27as) If ***Yes***, please, provide name of national reference lab: ____________________

27b) If ***Yes,*** were the results used to inform treatment schemes? ☐
1= Yes 2= No

28. Is surveillance of gonococcal antimicrobial susceptibility conducted in your country? ☐
1= Yes 2= No (***Go to*** Q29)

28a) Year of latest study of susceptibility testing: ☐☐☐☐
9999 if ***Don't know*** or ***Data not available***

28b) Name of test method: ____________________

PROJECT ID: A 6 5 9 9 6

Date form completed:

Day		Month		Year			
				2	0	1	9

Name of Country: ______________________

IX. HPV vaccination, screening, diagnosis, and treatment

29. Is the human papillomavirus (HPV) vaccine included in the national immunization schedule? ☐
1= Yes 2= No (***Go to*** Q30)

29a) If ***Q29= Yes*** , please provide the WEB link of National immunization schedule:

29a1) Provide WEB link if ***Available*** : ______________________
OR
29a2) Check the box if WEB link is ***Not available:*** ☐

29b) If ***Q29= Yes*** , to whom and at what age?

29b1) Adolescent girls: ☐
1= Yes 2= No

29b2) Adolescent girls and boys: ☐
1= Yes 2= No

29b3) Age range for HPV vaccine: ☐☐
99 if ***Don't know***

29b4) Is the human papillomavirus (HPV) vaccine for PLHIV included in the national immunization schedule? ☐
1= Yes 2= No

29b4a) If ***Yes,*** what is the age range for PLHIV: ☐☐
99 if ***Don't know***

29b5) Is the human papillomavirus (HPV) vaccine for MSM included in the national immunization schedule? ☐
1= Yes 2= No

29b5a) If ***Yes,*** what is the age range for MSM: ☐☐
99 if ***Don't know***

30. Does your country have a national cancer registry that includes cervical cancer? ☐
1= Yes 2= No (***Go to*** Q31)

	Yes	No
30a) Population based	☐	☐
30b) Hospital based	☐	☐
30c) National	☐	☐
30d) Sub-National	☐	☐

31. Is screening for cervical cancer available for general populations of women between the ages of 35 and 55? ☐
1= Yes 2= No (***Go to*** Q33)

PROJECT ID:

A	6	5	9	9	6

Date form completed:

Day		Month		Year			
				2	0	1	9

Name of Country: ______________________

IX. HPV vaccination, screening, diagnosis, and treatment *(Continued)*

32. If cervical cancer screening is available, which tests are used? **Yes** **No**

- 32a) PAP smear/test ☐ ☐
- 32b) HPV test ☐ ☐
  - 32bs) If ***HPV test=Yes*** , please provide which HPV test is used: ______________
- 32c) Acetic acid testing ☐ ☐
- 32d) Visual inspection only ☐ ☐
- 32e) Other ☐ ☐
  - 32es) If ***Other= Yes*** , please specify: ______________

33. What treatments are used for patients with abnormal cervical screening results (pre-cancerous lesions)?

Yes **No**

- 33a) Thermo ablation ☐ ☐
- 33b) Cryotherapy (liquid nitrogen) ☐ ☐
- 33c) Loop Electrosurgical Excision Procedure or conization ☐ ☐
- 33d) Surgical removal with other procedure ☐ ☐
- 33e) Other ☐ ☐
  - 33es) If ***Other= Yes*** , please specify: ______________

X - Technical assistance

34.Is the country in need to WHO technical assistance? ☐

1= Yes 2= No (***Go to*** Q31)

If ***Yes*** , on which areas? **Yes** **No**

- 34a) Development of National STI strategy ☐ ☐
- 34b) Development of National STI treatment guidelines ☐ ☐
- 34c) STI care services ☐ ☐
- 34d) STI surveillance ☐ ☐
- 34e) EMTCT of HIV and syphilis ☐ ☐
- 34f) Screening, diagnosis and treatment ☐ ☐
- 34g) Surveillance in AMR resistance in Gonorrhoea ☐ ☐
- 34h) HPV vaccination, screening, diagnosis and treatment ☐ ☐

35.What other technical partners / agencies are supporting you in the STI prevention and control services, and in which particular area? ______________

36.What is the main source of funding support for implementation of STI-related prevention and control programmes in country? ______________

(***Date and Sign*** questionnaire)

To be completed by Responder:

Name: ______________

Signature: ______________

Date:

Day		Month		Year			
				2	0	1	9

To be completed by Data Entry Operator:

Name: ______________

Signature: ______________

Date:

Day		Month		Year			
				2	0	1	9
