# Supplementary material for: Assessment of country implementation of the WHO global health sector strategy on sexually transmitted infections (2016-2021)
Source: PLoS One. 2022 May 4;17(5):e0263550. doi: 10.1371/journal.pone.0263550 (PMC9067912; doi:10.1371/journal.pone.0263550)
Supplement: S1 Table — (DOCX) [file pone.0263550.s002.docx]

**S1 Table: Countries responding to the WHO STI Global Health Sector Strategy Survey (N = 112)**

| **WHO Region** | | | | | |
| --- | --- | --- | --- | --- | --- |
| **African** | **Americas** | **South-East Asia** | **European** | **Eastern Mediterranean** | **Western Pacific** |
| Algeria | Argentina | Bhutan | Armenia | Egypt | Australia |
| Benin | Belize | Democratic Peoples Republic of Korea | Austria | Iran, Islamic Republic of | Brunei Darussalam |
| Burkina Faso | Bolivia | India | Belgium | Lebanon | Cambodia |
| Cameroon | Brazil | Indonesia | Croatia | Morocco | China |
| Central African Republic | Canada | Maldives | Cyprus | Oman | Cook Islands |
| Chad | Colombia | Myanmar | Denmark | Pakistan | Fiji |
| Congo | Costa Rica | Nepal | France |  | Japan |
| Cote d'Ivoire | Cuba | Sri Lanka | Georgia |  | Korea, Republic of |
| Democratic Republic of the Congo | Dominican Republic | Thailand | Germany |  | Lao People's Democratic Republic |
| Ethiopia | Ecuador | Timor-Leste | Greece |  | Malaysia |
| Gabon | El Salvador |  | Hungary |  | Mongolia |
| Gambia | Grenada |  | Ireland |  | New Zealand |
| Ghana | Guatemala |  | Kazakhstan |  | Papua New Guinea |
| Liberia | Guyana |  | Lithuania |  | Philippines |
| Madagascar | Honduras |  | Moldova, Republic of |  | Samoa |
| Mali | Jamaica |  | Monaco |  | Singapore |
| Namibia | Mexico |  | Norway |  | Solomon Islands |
| Niger | Nicaragua |  | Poland |  | Viet Nam |
| Nigeria | Panama |  | Portugal |  |  |
| Rwanda | Paraguay |  | Russian Federation |  |  |
| Sierra Leone | Peru |  | Serbia |  |  |
| South Africa | Saint Lucia |  | Slovenia |  |  |
| South Sudan | Trinidad and Tobago |  | Spain |  |  |
| Tanzania, United Republic of | United States of America |  | Sweden |  |  |
| Togo | Venezuela |  | Switzerland |  |  |
| Zimbabwe |  |  | Turkey |  |  |
|  |  |  | Ukraine |  |  |
