# Supplementary material for: Assessment of country implementation of the WHO global health sector strategy on sexually transmitted infections (2016-2021)
Source: PLoS One. 2022 May 4;17(5):e0263550. doi: 10.1371/journal.pone.0263550 (PMC9067912; doi:10.1371/journal.pone.0263550)
Supplement: S2 Table — (DOCX) [file pone.0263550.s003.docx]

**S2 Table: Survey response rates by WHO region and World Bank Income Classification**

|  | **Total** | **Survey completed** | | | |
| --- | --- | --- | --- | --- | --- |
|  |  | **No** | | **Yes** | |
|  | **N** | **N** | **%** | **N** | **%** |
| All | 194 | 82 | 42% | 112 | 58% |
| **WHO Region** |  |  |  |  |  |
| *African* | 47 | 21 | 45% | 26 | 55% |
| *Americas* | 35 | 10 | 29% | 25 | 71% |
| *South East Asia* | 11 | 1 | 9% | 10 | 91% |
| *European* | 53 | 26 | 49% | 27 | 51% |
| *Eastern Mediterranean* | 21 | 15 | 71% | 6 | 29% |
| *Western Pacific* | 27 | 9 | 33% | 18 | 67% |
| **World Bank Income Classification** |  |  |  |  |  |
| *High income* | 57 | 27 | 47% | 30 | 53% |
| *Upper middle income* | 58 | 22 | 38% | 36 | 62% |
| *Lower middle income* | 46 | 19 | 41% | 27 | 59% |
| *Low income* | 33 | 14 | 42% | 19 | 58% |
