# Supplementary material for: Assessment of country implementation of the WHO global health sector strategy on sexually transmitted infections (2016-2021)
Source: PLoS One. 2022 May 4;17(5):e0263550. doi: 10.1371/journal.pone.0263550 (PMC9067912; doi:10.1371/journal.pone.0263550)
Supplement: S3 Table — * Elimination of mother-to-child transmission of HIV and syphilis. **16 between 2020–2023. (DOCX) [file pone.0263550.s004.docx]

**S3 Table: National EMTCT* policy and programming by World Bank Income Classification**

| **EMTCT* programming**  **Countries reporting having strategy, policy or plan** | **All Responding Countries** | **High Income** | **Upper-Middle Income** | **Lower-Middle Income** | **Low Income** |
| --- | --- | --- | --- | --- | --- |
| National strategy for EMTCT | 70% (78/112) | 13/29 (45%) | 30/36 (83%) | 27/27 (78%) | 14/19 (74%) |
| National strategy only for HIV | 10% (11/110) | 3/29 (10%) | 1/36 (3%) | 3/27 (11%) | 4/19 (21%) |
| Plan to apply for validation of EMTCT or path to elimination of both HIV and syphilis | 74% (58/79) ** | 4/12 (33%) | 27/31 (87%) | 17/22 (77%) | 10/14 (71%) |
| National policy for screening pregnant women for HIV | 93% (104/108) | 27/30 (90%) | 36/36 (100) | 26/27 (96%) | 15/19 (79%) |
| National policy for screening pregnant women for syphilis | 93% (103/111) | 28/30 (93%) | 35/36 (97%) | 25/26 (96%) | 15/19 (79%) |

* Elimination of mother-to-child transmission of HIV and syphilis

**16 between 2020-2023
