# Supplementary material for: Assessment of country implementation of the WHO global health sector strategy on sexually transmitted infections (2016-2021)
Source: PLoS One. 2022 May 4;17(5):e0263550. doi: 10.1371/journal.pone.0263550 (PMC9067912; doi:10.1371/journal.pone.0263550)
Supplement: S4 Table — (DOCX) [file pone.0263550.s005.docx]

**S4 Table: National STI Surveillance System Elements by World Bank Income Classification**

| **STI Surveillance System Elements**  **Countries reporting surveillance system elements in place** | **All Responding Countries** | **High Income** | **Upper-Middle Income** | **Lower-Middle Income** | **Low Income** |
| --- | --- | --- | --- | --- | --- |
| STI surveillance or monitoring | 97/111 (87%) | 29/30 (97%) | 30/36 (83%) | 21/26 (81%) | 17/19 (90%) |
| STI surveillance integrated within the National Health Information System (NHIS) | 82/101 (81%) | 24/29 (83%) | 23/30 (77%) | 20/25 (80%) | 15/17 (88%) |
| STI case reporting | 96/98 (91%) | 28/29 (97%) | 30/30 (100%) | 22/22 (100%) | 16/17 (94%) |
| Syndromic |  |  |  |  |  |
| *Urethral discharge* | *48/95 (51%)* | *5/28 (18%)* | *16/29 (55%)* | *13/22 (59%)* | *14/16 (88%)* |
| *Genital ulcer disease* | *45/95 (47%)* | *5/28 (18%)* | *14/29 (48%)* | *14/22 (64%)* | *12/16 (75%)* |
| *Vaginal discharge* | *44/95 (46%)* | *3/28 (11%)* | *14/29 (48%)* | *13/22 (59%)* | *14/16 (88%)* |
| Etiologic |  |  |  |  |  |
| *Syphilis* | *71/96 (74%)* | *25/28 (89%)* | *24/30 (80%)* | *13/22 (59%)* | *9/16 (56%)* |
| *Gonorrhoea* | *58/95 (61%)* | *23/27 (85%)* | *19/30 (63%)* | *10/22 (45%)* | *6/16 (38%)* |
| *Chlamydia* | *42/96 (44%)* | *17/28 (61%)* | *15/30 (50%)* | *5/22 (23%)* | *5/16 (31%)* |
| *Trichomoniasis* | *30/96 (31%)* | *3/28 (11%)* | *13/30 (43%)* | *9/22 (41%)* | *5/16 (31%)* |
| Etiologic assessment of STI syndromes | 48/98 (48%) | 7/29 (24%) | 20/30 (67%) | 10/22 (45%) | 11/17 (65%) |
| Periodic STI prevalence surveys among general and high-risk populations | 49/97 (49%) | 12/29 (41%) | 14/30 (47%) | 17/22 (77%) | 5/16 (31%) |
