# Supplementary material for: Assessment of country implementation of the WHO global health sector strategy on sexually transmitted infections (2016-2021)
Source: PLoS One. 2022 May 4;17(5):e0263550. doi: 10.1371/journal.pone.0263550 (PMC9067912; doi:10.1371/journal.pone.0263550)
Supplement: S5 Table — (DOCX) [file pone.0263550.s006.docx]

**S5 Table: Laboratory antimicrobial resistance (AMR) surveillance by World Bank Income Classification**

| **AMR laboratory surveillance activity** | **All Responding Countries** | **High Income** | **Upper-Middle Income** | **Lower-Middle Income** | **Low Income** |
| --- | --- | --- | --- | --- | --- |
| Perform general antimicrobial susceptibility testing at national reference laboratory | 86/108 (80%) | 27/29 (93%) | 26/36 (72%) | 20/25 (80%) | 13/18 (72%) |
| Perform AMR testing for *N. gonorrhoeae* at national reference laboratory | 80/90 (89%) | 27/27 (100%) | 25/27 (93%) | 17/21 (81%) | 11/15 (73%) |
| Use results to inform gonorrhoea treatment recommendations | 66/84 (79%) | 23/27 (85%) | 20/23 (87%) | 14/20 (70%) | 9/14 (64%) |
| Surveillance of gonococcal antimicrobial susceptibility | 57/89 (64%) | 24/27 (89%) | 16/26 (62%) | 9/22 (41%) | 8/14 (57%) |
