# Supplementary material for: Assessment of country implementation of the WHO global health sector strategy on sexually transmitted infections (2016-2021)
Source: PLoS One. 2022 May 4;17(5):e0263550. doi: 10.1371/journal.pone.0263550 (PMC9067912; doi:10.1371/journal.pone.0263550)
Supplement: S6 Table — (DOCX) [file pone.0263550.s007.docx]

**S6 Table: National human papillomavirus virus (HPV) vaccine schedules by World Bank Income Classification**

| **HPV vaccine** | **All Responding Countries** | **High Income** | **Upper-Middle Income** | **Lower-Middle Income** | **Low Income** |
| --- | --- | --- | --- | --- | --- |
| Included in the national immunization schedule | 65/110 (59%) | 28/30 (93%) | 23/26 (64%) | 8/25 (32%) | 6/19 (32%) |
| Recommended for adolescent girls | 63/67 (94%) | 27/28 (96%) | 22/23 (96%) | 8/9 (89%) | 6/7 (86%) |
| Recommended for adolescent girls and boys | 20/65 (31%) | 14/14 (100%) | 5/22 (23%) | 0/8 (0%) | 1/7 (14%) |
| Recommended for people living with HIV | 18/68 (26%) | 9/28 (32%) | 3/24 (13%) | 1/9 (11%) | 2/7 (29%) |
| Recommended for men who have sex with men | 12/68 (18%) | 9/28 (32%) | 3/24 (13%) | 0/9 (0%) | 0/7 (0%) |
