# Supplementary material for: Assessment of country implementation of the WHO global health sector strategy on sexually transmitted infections (2016-2021)
Source: PLoS One. 2022 May 4;17(5):e0263550. doi: 10.1371/journal.pone.0263550 (PMC9067912; doi:10.1371/journal.pone.0263550)
Supplement: S1 Checklist — (DOCX) [file pone.0263550.s008.docx]

***PLOS ONE* Clinical Studies Checklist**

***PLOS ONE* manuscript number: _** **PONE-D-22-01956**

| **Complete the following if your study involved human participants or human subjects’ data. These questions should be addressed for prospective and retrospective studies.** | | |
| --- | --- | --- |
| 1. | Did you obtain ethics approval for this study?   - If yes, please upload (file type “Other”) the original approval document you received from your ethics committee. If the original document is in another language, please also provide an English translation.   ___ Uploaded ___ N/A   - If you did not obtain ethical approval, please explain why this was not required.  \| Thank you. We appreciate your attention to this information and appreciate your review of our proposed language.  This survey of STI program representatives was undertaken for public health and program surveillance to evaluate the implementation of the WHO STI Strategy. According to the Common Rule section (5)(iii): <https://www.hhs.gov/ohrp/regulations-and-policy/regulations/45-cfr-46/common-rule-subpart-a-46104/index.html>. evaluations of public health implementation and programming of this type are not considered human subjects research as they are for "public health activities and purposes" as described under 45 CFR 164.512(b); . <https://www.law.cornell.edu/cfr/text/45/164.512> “**(i)** A [public health authority](https://www.law.cornell.edu/definitions/index.php?width=840&height=800&iframe=true&def_id=e7505cb5be9f8d4841d34694772af9ae&term_occur=999&term_src=Title:45:Chapter:A:Subchapter:C:Part:164:Subpart:E:164.512) that is authorized by law to collect or receive such information for the purpose of preventing or controlling disease, injury, or disability, including, but not limited to, the reporting of disease, injury, vital events such as birth or death, and the conduct of public health surveillance, public health investigations, and public health interventions; or, at the direction of a [public health authority](https://www.law.cornell.edu/definitions/index.php?width=840&height=800&iframe=true&def_id=e7505cb5be9f8d4841d34694772af9ae&term_occur=999&term_src=Title:45:Chapter:A:Subchapter:C:Part:164:Subpart:E:164.512), to an official of a foreign government agency that is [acting](https://www.law.cornell.edu/definitions/index.php?width=840&height=800&iframe=true&def_id=0428940761849f2241da1c4964c44de8&term_occur=999&term_src=Title:45:Chapter:A:Subchapter:C:Part:164:Subpart:E:164.512) in collaboration with a [public health authority](https://www.law.cornell.edu/definitions/index.php?width=840&height=800&iframe=true&def_id=e7505cb5be9f8d4841d34694772af9ae&term_occur=999&term_src=Title:45:Chapter:A:Subchapter:C:Part:164:Subpart:E:164.512);”  No personal identifying information of the respondents was used. Respondents were made aware that the information they provided would be used to gauge implementation of the WHO STI strategy at region and global levels. Participants names were removed during data entry and analysis and data were not reported at the country level. We will enter this into the methods. Survey respondents consented to the collection and use of the survey information for regional, and global evaluation of the implementation of the WHO STI Strategy. \| \| --- \| |  |
| 2. | If your study involved human participants, please report in the Methods section when participants were recruited to the study.  ___ Completed __X_ N/A |  |
| 3. | If you are reporting a study of medical records or archived samples, please report in the Methods section the date range in which human subjects’ data/samples were collected and the date(s) when you conducted this study.  ___ Completed _X__ N/A |  |
| 4. | Please specify in the Methods section whether authors had access to information that could identify individual participants during or after data collection.  __X_ Completed ___ N/A |  |
| 5. | If you are reporting an observational study – i.e. cohort, case-control, and cross-sectional studies – we recommend that the work is reported as per the requirements of the STROBE guidelines, and that you provide a completed STROBE checklist as a Supporting Information file with your submission.  The STROBE checklist was developed to improve the reporting of observational human subjects research, and is available here: <http://strobe-statement.org/fileadmin/Strobe/uploads/checklists/STROBE_checklist_v4_combined_PlosMedicine.docx>.  ___ Completed X___ N/A |  |
| 6. | Please ensure that the author list and Corresponding Author entered in Editorial Manager match the author list and Corresponding Author in your manuscript file.  __X_ Completed |  |
